# Intrahepatic Cholangiocarcinoma Presenting as an Incidentaloma on F-18 PYL PSMA PET/CT

**DOI:** 10.1055/s-0044-1793833

**Published:** 2024-11-13

**Authors:** Rishi R. Patel, Udhayvir Singh Grewal, Yiqin Xiong, Naomi Fei

**Affiliations:** 1 Carver College of Medicine, University of Iowa, Iowa City, Iowa, United States; 2 Division of Hematology, Oncology, and Blood and Marrow Transplantation, University of Iowa Hospitals and Clinics, Iowa City, Iowa, United States; 3 Department of Pathology, University of Iowa Hospitals and Clinics, Iowa City, Iowa, United States

**Keywords:** cholangiocarcinoma, incidentaloma, intrahepatic cholangiocarcinoma, PSMA PET/CT, theranostics

## Abstract

Cholangiocarcinoma is an aggressive malignancy arising from the biliary tract epithelium with rising incidence and mortality. Imaging commonly used for diagnostic workup includes computed tomography (CT) scan and magnetic resonance imaging (MRI). Fluorine-18 fluorodeoxyglucose positron emission tomography (FDG PET) scans can be used for investigating equivocal findings on radiographic imaging. Prostate-specific membrane antigen (PSMA) is known to be highly expressed in prostate adenocarcinoma, allowing it to be leveraged as a target for both imaging and radioligand therapy in prostate cancer. However, PSMA is also commonly expressed in other malignancies such as breast cancer, thyroid carcinomas, and head and neck malignancies, which increase the chances of their incidental diagnosis on PSMA PET scans. Biliary tract cancers, including cholangiocarcinoma, are not commonly expected to be incidentally diagnosed on PSMA PET imaging. Here, we describe the case of a patient with known prostate adenocarcinoma who was later incidentally diagnosed with advanced intrahepatic cholangiocarcinoma on PSMA PET/computed tomography (CT). This case highlights the unexpected finding of cholangiocarcinoma on PSMA PET/CT and suggests further investigation into the role of PSMA PET in diagnosing ambiguous cases of cholangiocarcinoma and its potential theranostic applications.

## Introduction

Cholangiocarcinoma is an aggressive malignancy arising from the biliary tract epithelium with rising incidence and mortality. 1 Imaging commonly used for diagnostic workup include computed tomography (CT) scan and magnetic resonance imaging (MRI). Fluorine-18 (F-18) fluorodeoxyglucose positron emission tomography (FDG PET) scans can be used for investigating equivocal findings on radiographic imaging. Prostate-specific membrane antigen (PSMA) is known to be highly expressed in prostate adenocarcinoma allowing it to be leveraged as a target for both imaging and radioligand therapy in prostate cancer. However, PSMA is also commonly expressed in other malignancies such as breast cancer, thyroid carcinomas, and head and neck malignancies, which increases the chances of their incidental diagnosis on PSMA PET scans. 2 3 4 However, biliary tract cancers are not commonly expected to be incidentally diagnosed on PSMA PET imaging. Here, we describe the case of a patient with known prostate adenocarcinoma who was later incidentally diagnosed with advanced intrahepatic cholangiocarcinoma on PSMA PET/CT.

## Case Report

**Fig. 1:**
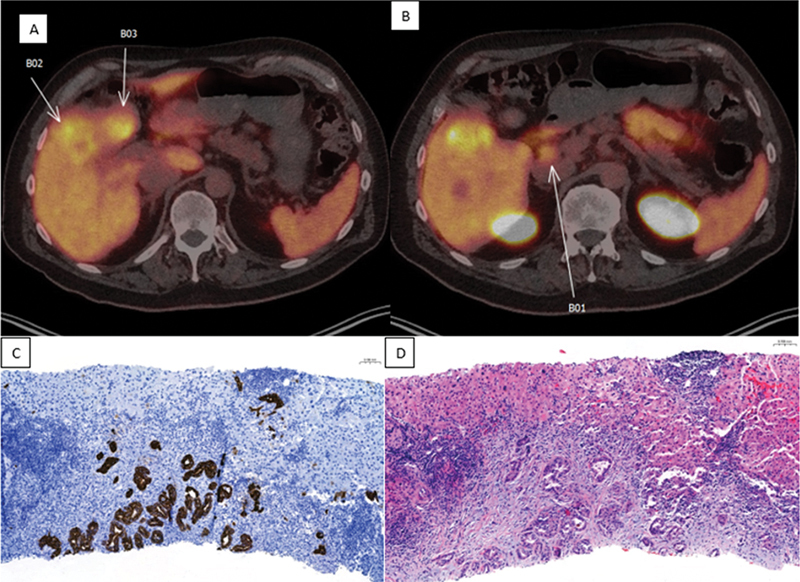
Fluorine-18 (F-18) PYL prostate-specific membrane antigen positron emission tomography/computed tomography cross-sectional image showing ( **A** ) tracer-avid lesions in segment 4B/5 suggestive of primary malignancy (B02, B03) and ( **B** ) tracer-avid lesion indicative of metastatic spread to portocaval lymph node (B01). Histopathological examination demonstrating ( **C** ) focus of strong CK7 positivity on immunohistochemistry and ( **D** ) hematoxylin and eosin stain demonstrating well-differentiated adenocarcinoma.

A 66-year-old man with past medical history of prostate cancer, thyroid cancer, hypertension, and atrial fibrillation underwent F-18 PYL PSMA PET/CT for staging of localized prostate adenocarcinoma prior to scheduled radical prostatectomy. The F-18 PYL PSMA PET/CT showed a large area of heterogenous PYL uptake (SUV _max_ = 16.9) involving liver segments 4B and 5 that was suspicious for a primary liver malignancy ( Fig. 1A ). Follow-up MRI revealed a 5.8 × 4.5 cm segment 4B/5 hepatic mass with adjacent gallbladder invasion and enlarged portacaval lymph node suggestive of metastatic disease. Ultrasound-guided needle biopsy of the liver was noted to be consistent with a well-differentiated adenocarcinoma. Immunostaining was positive for CK7 and negative for HepPar 1, NKX3.1, and TTF-1, favoring a diagnosis of cholangiocarcinoma ( Fig. 1C ). The patient underwent staging imaging with CT of the chest, abdomen, and pelvis, which revealed metastatic disease involving the anterior abdominal wall. The patient was treated for metastatic intrahepatic cholangiocarcinoma with standard of care chemoimmunotherapy (gemcitabine, cisplatin, and durvalumab). He remains in follow-up on maintenance immunotherapy.

## Discussion

In the current report, we present a case of intrahepatic cholangiocarcinoma found incidentally on a F-18 PYL PSMA PET scan. As discussed earlier, cholangiocarcinoma is not typically expected to be incidentally diagnosed on a PSMA PET/CT.

On literature review, we found previous studies noting a high rate of positivity for PSMA on immunohistochemistry in cholangiocarcinoma. An analysis of 203 samples of cholangiocarcinoma found that 79.3% of samples were PSMA positive on immunohistochemistry. In addition, PSMA expression positively correlated with the grade and stage of cholangiocarcinoma. The PSMA positive cases were seen more in stages III and IV and high-grade disease compared with early-stage and low-grade disease, respectively. 5 A few prior case reports of intrahepatic cholangiocarcinoma detected PSMA PET imaging have been reported. 6 7 8 There are insufficient data to determine if PSMA PET can be reliably used to differentiate between metastases from prostate cancer and primary hepatobiliary malignancies. However, there should be a high index of suspicion for the latter, especially in the presence of multiple risk factors that increase the risk of primary hepatobiliary malignancies.

The mechanism of PSMA expression tends to differ between malignant tumors such as primary hepatobiliary malignancies and benign liver lesions. PSMA is primarily expressed on neovascular endothelium as opposed to increased expression by virtue of increased blood flow or folate receptors in macrophages in the case of benign liver lesions. 5 Interestingly, a recent analysis of 72 liver tumors also identified perivascular PSMA as a potential biomarker for distinguishing between primary cholangiocarcinoma and metastatic pancreatic adenocarcinoma, two diagnoses that are commonly difficult to distinguish by histology alone. 9 Altogether, these results suggest that PSMA PET merits further investigation for potential application in the diagnosis of unclear cases of cholangiocarcinoma. Furthermore, these findings may further serve as the basis for investigating the role of theranostic applications of PSMA expression in cholangiocarcinoma.

## References

[1] Gad M M Saad A M Faisaluddin M Epidemiology of cholangiocarcinoma; United States incidence and mortality trends Clin Res Hepatol Gastroenterol 2020 44 06 885 893 32359831 10.1016/j.clinre.2020.03.024

[2] Bertagna F Albano D Giovanella L ^68^ Ga-PSMA PET thyroid incidentalomas Hormones (Athens) 2019 18 02 145 149 30989578 10.1007/s42000-019-00106-8

[3] Zhou W Halder S Herwald S Frequent amplification and overexpression of PSMA in basallike breast cancer from analysis of The Cancer Genome Atlas J Nucl Med 2024 65 07 1004 1006 38664014 10.2967/jnumed.123.266659

[4] Lawhn-Heath C Flavell R R Glastonbury C Hope T A Behr S C Incidental detection of head and neck squamous cell carcinoma on ^68^ Ga-PSMA-11 PET/CT Clin Nucl Med 2017 42 04 e218 e220 28166149 10.1097/RLU.0000000000001569

[5] Chen L X Zou S J Li D Prostate-specific membrane antigen expression in hepatocellular carcinoma, cholangiocarcinoma, and liver cirrhosis World J Gastroenterol 2020 26 48 7664 7678 33505143 10.3748/wjg.v26.i48.7664 PMC7789058

[6] Kang C Jiang J Y Lee M E Shen L Mansberg R Incidental intrahepatic hepatocellular cholangiocarcinoma detected on ^68^ Ga-PSMA PET/CT Clin Nucl Med 2022 47 03 e291 e293 35020661 10.1097/RLU.0000000000003992

[7] Veenstra M MK Vegt E Segbers M Intra-arterial PSMA injection using hepatic arterial infusion pump in intrahepatic cholangiocarcinoma: a proof-of-concept study Eur Radiol Exp 2024 8 01 90 39090480 10.1186/s41747-024-00496-4 PMC11294287

[8] Sun Y Wang H Yang Y You Z Zhao J Intrahepatic cholangiocarcinoma detected on ^18^ F-PSMA-1007 PET/MR imaging in a prostate cancer patient: a case report and literature review Front Oncol 2024 14 1.408453E6 10.3389/fonc.2024.1408453 PMC11199518 38933442

[9] Chen W Lee Z Awadallah A Zhou L Xin W Peritumoral/vascular expression of PSMA as a diagnostic marker in hepatic lesions Diagn Pathol 2020 15 01 92 32703222 10.1186/s13000-020-00982-4 PMC7376868

